# Label propagation defines signaling networks associated with recurrently mutated cancer genes

**DOI:** 10.1038/s41598-019-45603-3

**Published:** 2019-06-28

**Authors:** Merve Cakir, Sayan Mukherjee, Kris C. Wood

**Affiliations:** 1 Program in Computational Biology and Bioinformatics, Duke University, Durham, NC 27708 USA; 2 Department of Pharmacology and Cancer Biology, Duke University, Durham, NC 27710 USA; 3 Departments of Statistical Science, Mathematics, Computer Science, Biostatistics & Bioinformatics, Duke University, Durham, NC 27708 USA

**Keywords:** Cellular signalling networks, Systems biology, Bioinformatics, Cancer genomics

## Abstract

Human tumors have distinct profiles of genomic alterations, and each of these alterations has the potential to cause unique changes to cellular homeostasis. Detailed analyses of these changes could reveal downstream effects of genomic alterations, contributing to our understanding of their roles in tumor development and progression. Across a range of tumor types, including bladder, lung, and endometrial carcinoma, we determined genes that are frequently altered in The Cancer Genome Atlas patient populations, then examined the effects of these alterations on signaling and regulatory pathways. To achieve this, we used a label propagation-based methodology to generate networks from gene expression signatures associated with defined mutations. Individual networks offered a large-scale view of signaling changes represented by gene signatures, which in turn reflected the scope of molecular events that are perturbed in the presence of a given genomic alteration. Comparing different networks to one another revealed common biological pathways impacted by distinct genomic alterations, highlighting the concept that tumors can dysregulate key pathways through multiple, seemingly unrelated mechanisms. Finally, altered genes inducing common changes to the signaling network were used to search for genomic markers of drug response, connecting shared perturbations to differential drug sensitivity.

## Introduction

Recent advances in high-throughput sequencing technologies and large-scale efforts like The Cancer Genome Atlas (TCGA) have revealed, for the first time, the landscapes of genomic alterations found within distinct tumor types, providing new insights into the mechanisms of tumor development and progression^1–3^. Certain genes, like *PIK3CA* and *TP53*, are mutated at a high frequency across a number of different tumors, whereas there are other genes whose mutations are only observed in a single tumor type. Further, comparisons of the patterns of genomic alterations within and across tumor types^4,5^ have revealed that tumors regulate their growth and survival through both shared and distinct mechanisms. Together, these studies underscore the importance of diverse and incompletely understood genomic changes in dictating tumors’ biological and therapeutic response characteristics.

The next step following identification of tumor genomic alterations is to understand how these alterations disturb cellular homeostasis or contribute to tumorigenesis. Connecting recurrently altered genes to the signaling or regulatory pathways they participate in is a common starting point to achieve this goal. For instance, a common approach employed by the TCGA and related projects is to place genes into groups representative of the pathways or processes in which they are believed to function, such as MAPK signaling, PI3K signaling, and cell cycle. However, in many cases, the alteration of a cancer gene leads to cellular changes that are only partially understood and cannot be easily placed into defined pathways. Additionally, even those genomic alterations involving genes canonically implicated in a defined pathway have the potential to induce non-canonical signaling changes. Therefore, grouping genomic alterations based only on our current knowledge-based annotations can lead to an incomplete representation of their effects. Alternative approaches that enable a more comprehensive look into signaling changes can provide a more in-depth understanding of downstream effects of frequently altered cancer genes. Further, analyzing these effects across different genes may highlight common signaling events that are perturbed downstream of distinct alterations, revealing the mechanisms by which tumors achieve common outcomes (e.g., growth and survival) via distinct mechanisms.

One way of performing a detailed analysis of the effects of defined genomic alterations on signaling events is by focusing on gene expression patterns to identify genes which display dysregulated expression in the presence of a given alteration. Creating a gene expression signature by comparing mutant and wild-type samples is an established method for such an analysis. This signature, however, will often result in a sparse representation of the molecular changes associated with an alteration, as it will typically be based on strong discriminators and cannot possibly contain every gene in a dysregulated pathway. Searching for enriched Gene Ontology or functional annotation terms is a common way to better understand the molecular events represented by a given gene expression signature^6,7^. Gene Set Enrichment Analysis (GSEA) is another commonly used method for connecting gene expression patterns to perturbations in signaling events^8^. One drawback of these enrichment based approaches, however, is the fact that they ignore connectivity within and between enriched gene sets. Thus, they yield information on the molecular events that are dysregulated but do not provide information on how the genes that contribute to these events functionally relate to one another. Additionally, these approaches lack information on the cross-talk between different gene sets that are dysregulated, making it harder to gain a unified understanding of the complete set of changes that are induced by a given genomic perturbation.

The connectivity within a given gene set, and crosstalk between different sets, offers valuable information because molecular events in a cell occur through an interconnected web of interactions. The changes that occur as a result of a given genomic alteration will spread across the molecular network through signaling cascades, rather than distinctly affecting separate sets of genes. Therefore, a more informative approach would be to create network-level views of signaling changes that are observed in the presence of a given alteration. This requires an approach that can go from a gene list level to a network structure that connects these genes. There are multiple ways of achieving this. For example, one can simply connect genes to their direct interaction partners found within the gene set or introduce intermediate genes that are found along the shortest paths connecting the elements of the gene set^9,10^.

Here, we have chosen instead to focus on another alternative, label propagation. Label propagation algorithms start with a given set of seed genes and diffuse through the network based on its specific topology to identify additional genes that are in the neighborhood of the seed genes, connecting them together. This diffusive property enables the algorithm to fully exploit the topological information offered by a given network and to discover a variety of paths that can connect a given gene set. This can create a more expansive representation of the starting gene set compared to linking direct neighbors or connecting them only through shortest paths. In biology, label propagation algorithms have been used to address several different problems, such as predicting functions of genes based on their relationships with other well-annotated genes^11^, discovering novel genes that are associated with a disease^12,13^, or differentiating potential driver mutations from passengers^14^. In cancer genomics, label propagation or related diffusion-based processes have been used to identify subnetworks that are populated by genes frequently mutated in patients, revealing signaling events that are significantly enriched for genes with frequent alterations^15,16^ or enabling stratification of patients based on similarity profiles of significantly mutated subnetworks^17^. These various applications of label propagation algorithm highlight its potential in discovering biologically meaningful interactions between a set of genes.

In this study, we used a label propagation-based methodology to create networks of signaling changes that are observed downstream of common oncogenic genomic alterations across different tumor types, with a particular emphasis on genes with recurrent mutations. Gene expression signatures, consisting of genes differentially expressed when a given gene is mutated, were used as seeds in a label propagation algorithm to explore a network of known signaling and regulatory pathways. The resulting network of each individual gene represents the range of molecular events that are dysregulated, revealing many of the specific signaling and regulatory pathways that are perturbed in connection to the genomic alterations in this particular gene. Comparing networks associated with different genes highlighted similarities in signaling pathways that were observed downstream of distinct alterations, revealing previously unappreciated convergence between the genes that drive cancer and highlighting cases where seemingly disparate mutations lead to common drug sensitivities.

## Results

### Label propagation creates networks from gene sets

**Figure 1:**
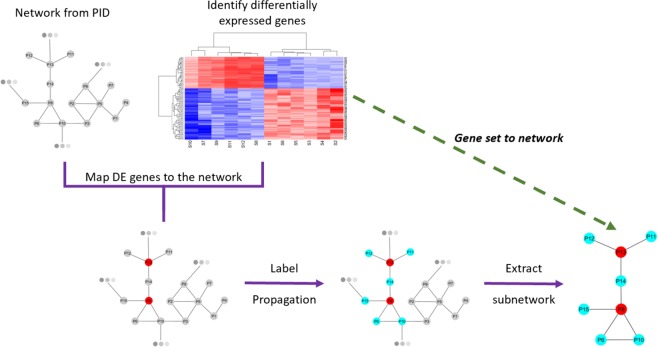
Description of the workflow. The analysis starts with identifying a set of differentially expressed genes (or any gene signature of interest) and obtaining a network of known biological interactions. Label propagation is run starting from the nodes in the network corresponding to genes in the signature to generate a subnetwork of interactions connecting these genes to additional functionally related genes, revealing signaling events represented by the gene set.

Label propagation-based approaches have the potential to fill in the missing links between a set of genes based on the connectivity information provided by a network. In this study, this algorithm was used to create networks of signaling and regulatory pathways starting from a set of genes representing a biological state. This set - which will also be referred to as seed gene set - can be any set of genes one is interested in analyzing in more detail, such as gene expression signatures predicting patient prognosis, a group of genes correlating with drug response, or a set of differentially expressed genes that can discriminate one phenotypic group from another. The second required input is a graph of biological interactions, where nodes represent genes and edges represent functional relationships between them and NCI’s Pathway Interaction Database (PID)^18^ was used in this workflow. After mapping the seed gene set to this network, an iterative diffusion process starts from seed genes, spreading information to the neighboring genes following the paths imposed by the network’s structure. The end result is a subnetwork that contains the seed gene set and the additional genes that are reached through the propagation process. The initial gene set will typically be a collection of genes sparsely representing a biological state and the resulting subnetwork fills in the blanks and provides a more expansive look into the molecular events represented by the gene set. Therefore, through this workflow, we are expanding a gene set into a network of functional interactions, connecting the biology represented with the gene set to relevant functional consequences. Additionally, we use a distance metric based on the idea of maximal common subgraph^19^ to compute pairwise distances between different networks. Smaller distances and higher similarity observed between networks reveal the set of networks that contains overlapping molecular events, which helps us discover unexpected connections between different gene sets, whereas high distances between networks reflect the sets of genes that represent distinct signaling pathways. Figure 1 provides a conceptual summary of this workflow, and implementation details are provided in the Methods section and associated Figures.

### Label propagation recovers pathways

**Figure 2:**
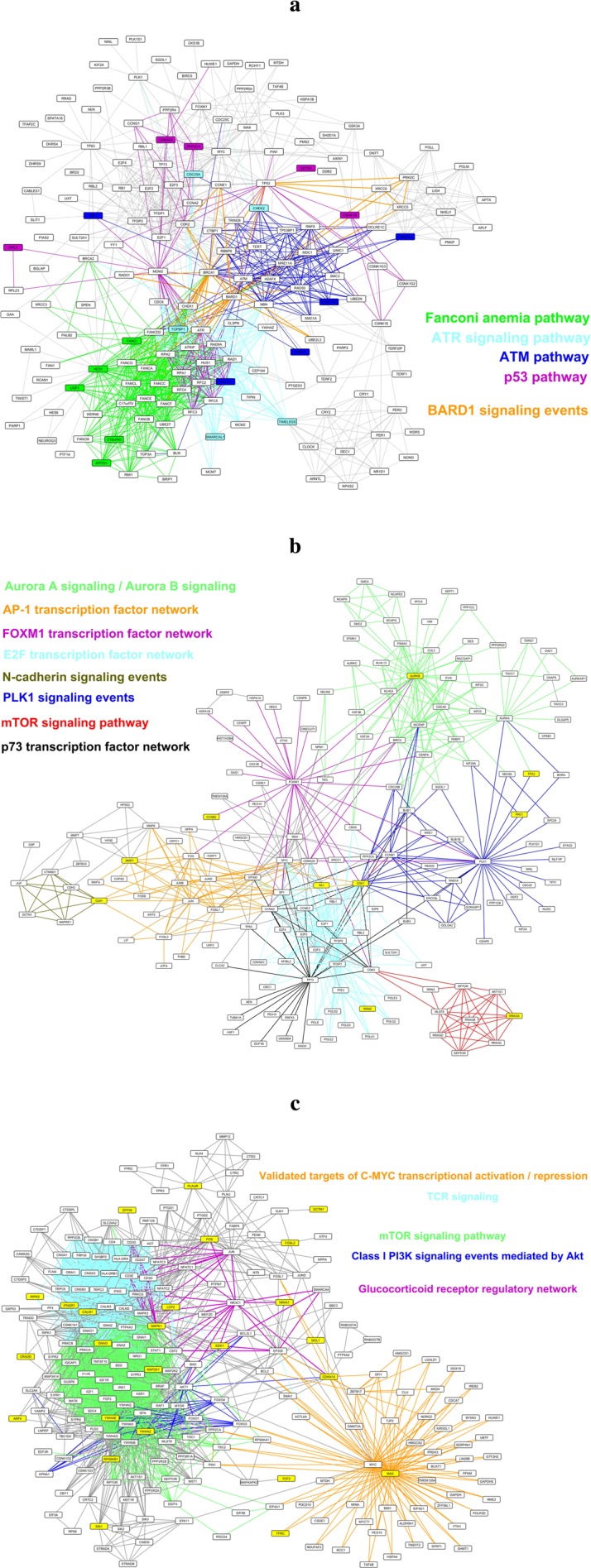
A variety of seed gene sets were used to test the workflow’s performance. In all three cases, edges belonging to different signaling events are color-coded and corresponding pathway names are listed next to the networks. (**a**) A set of genes belonging to four different pathways were randomly selected. The analysis filled in the missing parts of these pathways and additionally highlighted functionally related signaling events. Seed genes are color-coded based on the pathways to which they belong. (**b**) Genes from a “tamoxifen recurrence” signature were used as seed genes to create a subnetwork highlighting how this approach can be used to improve functional annotations of a given set of genes. Genes from the signature are shown in yellow. (**c**) Genes from a “glucocorticoid resistance” signature were used as seed genes to identify signaling pathways associated with the resistance phenotype. Genes from the signature are shown in yellow.

After establishing the workflow, we used a variety of seed gene sets to assess if this approach can successfully connect a particular gene set to the relevant biological pathways that it represents and generate a more expansive view of the molecular state that is sparsely represented by the gene set. The first case focused on a controlled set of seed genes, in order to test if the algorithm is capable of identifying multiple distinct pathways when the seed gene set contains genes representing a mixture of pathways. To form this set, we picked four individual pathways that have roles in DNA damage response and repair – namely “Fanconi anemia pathway”^20^, “ATR signaling pathway”^21^, “ATM pathway”^21^, and “p53 pathway”^22^. Five genes were randomly selected from each pathway to create a seed gene set containing 20 genes. The subnetwork obtained at the end of this label propagation run is shown in Fig. 2a. As can be seen, when starting with a gene set containing elements from multiple different pathways, the algorithm can recover parts of each individual pathway. In addition to the four pathways making up the seed gene set, the network in Fig. 2a contains the “BARD1 signaling events” pathway, which is also known to have a role in DNA damage response^23^. This means that the algorithm not only recovers missing parts of separate pathways represented by the seed gene set but also links these pathways to additional related pathways with which they interact, enabling a more holistic visualization of the molecular mechanisms that are sparsely represented by the starting gene set.

In most applications of this workflow, the participants of the seed gene set will be dictated by the biological phenomenon that one aims to summarize through a signature, and it will be a mixture of pathways with uneven numbers of genes belonging to each, in contrast to the first gene set with equal number of representatives from different pathways. To perform a test under this condition, we used a signature that predicts recurrence in breast cancer patients following treatment with the selective estrogen receptor modulator (SERM) tamoxifen^24^. This “tamoxifen signature” contains 36 genes, 10 of which are found in the PID network. These 10 genes were used as the seed genes to run the label propagation algorithm, and the resulting network is visualized in Fig. 2b. There is a substantial agreement between the functional roles assigned to these genes by Chanrion *et al*. and signaling events each seed gene was associated with in the resulting network (Supplementary Table S1). For instance, 5 out of these 10 genes have roles in mitosis and cell cycle and they all are represented here as parts of pathways that have well-established roles in mitotic machinery or cell cycle regulation, such as “Aurora A/B signaling”^25,26^, “FOXM1 transcription factor network”^27^, and “PLK1 signaling events”^28^. This concordance highlights that the label propagation approach was successful in linking these genes to biological pathways that are in line with their functional annotations and in discovering meaningful functional connections between these ten genes by localizing them to connected pathways.

We next examined if the expanded network view generated by this workflow offers additional insight into the biology summarized by a gene signature that may not be captured when solely focusing on the gene set itself. We used a gene signature generated by Wei *et al*., which represents correlates of resistance to glucocorticoid induced apoptosis in acute lymphoblastic leukemia (ALL)^29^. Genes in this signature were mapped to the PID network to be used as the seed genes and the resulting network is shown in Fig. 2c. Using the Connectivity Map (CMAP)^30^ and Gene Set Enrichment Analysis (GSEA)^8^, the authors suggested that the PI3K/Akt/mTOR signaling axis has a role in this resistance. Replicating the original study’s findings, the network contains genes and interactions belonging to “mTOR signaling pathway” and “Class I PI3K signaling events mediated by Akt”. The network, however, also contains genes that belong to additional signaling pathways not highlighted by the study. A more detailed look into the individual pathways represented in the network underscores the possibility of these events contributing to the resistance as some of these pathways, including “TCR signaling”, “Validated targets of C-MYC transcriptional activation”, and “Validated targets of C-MYC transcriptional repression”, have already been shown to have roles in regulating glucocorticoid induced apoptosis^31–35^. Additionally, a study by Da Costa *et al*. proposed treatment with the BET bromodomain inhibitor JQ1, an inhibitor of *MYC* transcription, as a way to sensitize ALL cells to dexamethasone treatment^36^. We next tested whether identification of these events is unique to our workflow or if they can be identified through a commonly used enrichment analysis. We used Enrichr^6^ to identify PID pathways that are enriched in the original signature and pathways shown in Fig. 2c, such as “TCR signaling” and *MYC* related signaling events, were not found to be significantly enriched in this signature (Supplementary Fig. S1). This implies that the described workflow recovered additional, functionally relevant pathways that are not otherwise apparent, highlighting the value of expanded network obtained through propagation. Together, the three cases shown in Fig. 2 demonstrate that the workflow is effective in converting a gene set to a signaling network composed of interactions corresponding to their functional roles, generating a broader view of the molecular events represented by the gene set.

### Tumors converge on select signaling pathways downstream of distinct genes

Following establishment and characterization of the workflow, we focused on studying the sets of genes frequently mutated across a range of tumor types to better understand molecular events dysregulated downstream of genes recurrently altered in cancer. We picked three different tumor types that have at least ten different genes frequently mutated in the TCGA sample set - urothelial bladder carcinoma, lung adenocarcinoma, and endometrial carcinoma. The following sections focus on the analyses of these genes with the label propagation approach and how we used pairwise distances between networks to highlight signaling events tumors converge on through genomic alterations in distinct genes.

#### Networks associated with genes recurrently mutated in bladder carcinoma

**Figure 3:**
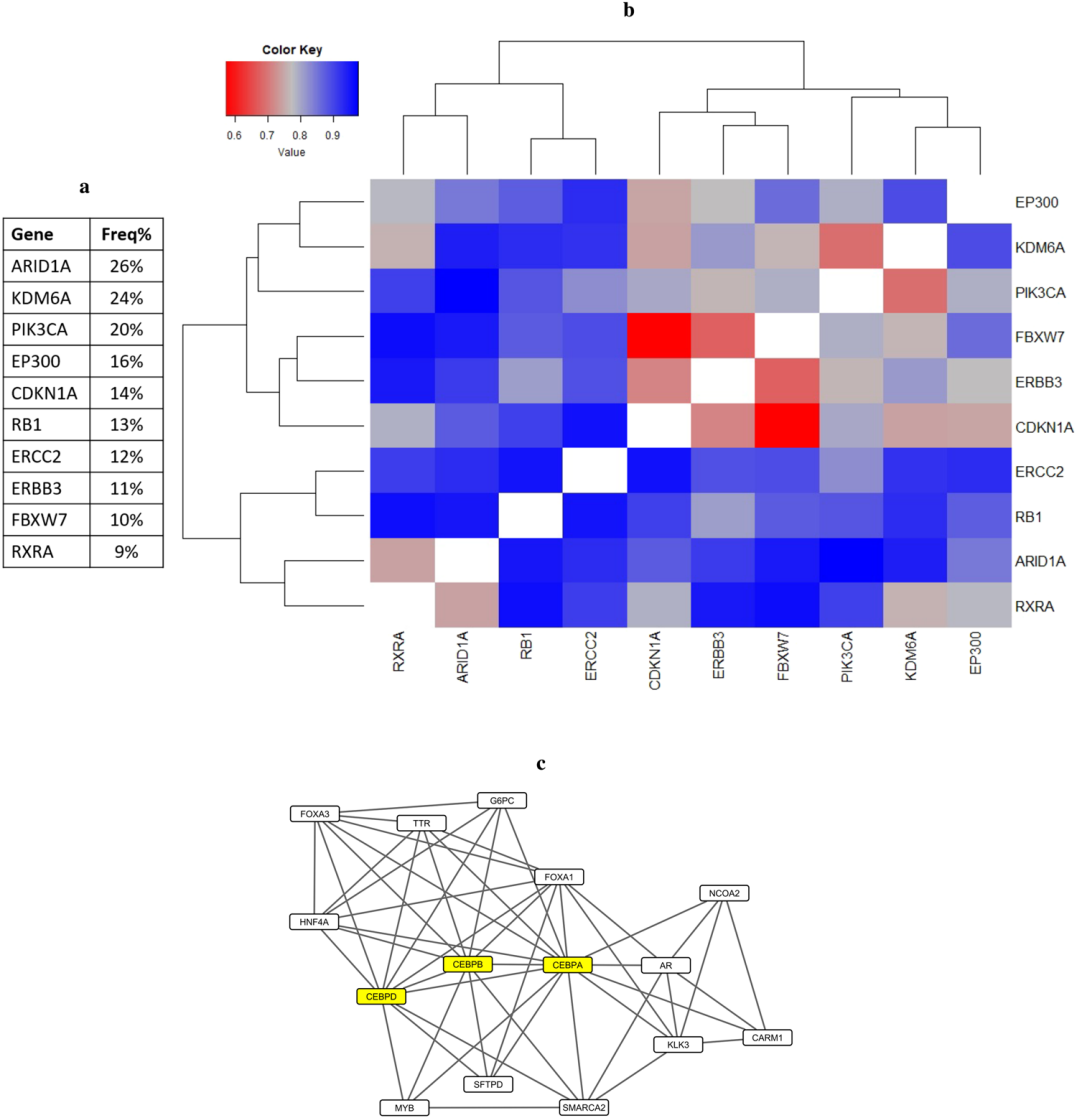
Signaling networks associated with frequently mutated genes in bladder carcinoma. (**a**) List of genes included in this analysis and their mutation frequency in the TCGA population. (**b**) Hierarchical clustering of the matrix representing pairwise distances between label propagation-based networks of genes frequently mutated in bladder carcinoma. Color code is shown in the upper left corner, where red corresponds to smaller distance values and blue corresponds to higher distance values. (**c**) A part of the maximal common subgraph of FBXW7- and CDKN1A-associated networks. The three genes with roles in cell cycle regulation are highlighted in yellow.

The first tissue type that we focused on was bladder carcinoma, as we investigated how the variety of alterations observed in this tumor type contributed to the dysregulation of key signaling events. As the first step of this analysis, we curated a list of genes that are identified to be significantly mutated in at least ~10% of bladder carcinoma patients by the TCGA study^1^, the study by Kandoth *et al*.^4^, and TumorPortal^5^ (Fig. 3a). For each gene within this list, we created an individual signaling subnetwork based on the workflow detailed in Fig. 1. A gene signature composed of genes differentially expressed when the gene of interest is mutated formed the seed gene set for label propagation. As a result, we obtained a set of networks revealing the underlying biology associated with the dysregulated genes, highlighting putative downstream effects of the selected altered genes. Additionally, to survey the relationships between these networks in a quantitative manner, we used the distance metric described in Methods section to compute the distances between every pair of networks and generate a pairwise distance matrix. To reveal the patterns of similarity, we performed hierarchical clustering on this matrix and the resulting heatmap is shown in Fig. 3b.

One pattern that this heatmap emphasizes is the fact that within the context of signaling pathways that were analyzed, the majority of networks do not share substantial similarities with others. This implies that distinct genomic alterations have non-overlapping downstream consequences that affect disparate parts of the molecular machinery. The rare cases where we do observe similarity then become more intriguing as these cases have the potential to reveal common downstream signaling events that are dysregulated through more than one mutational mechanism. For instance, the most similar pair in bladder carcinoma set is formed by the networks of FBXW7 and CDKN1A. This similarity cannot simply be explained by a high co-occurrence of *FBXW7* and *CDKN1A* mutations, as the frequency of patients with mutations in both genes is only 1.5%. Additionally, a follow-up analysis was performed, where samples mutant in *FBXW7* (or *CKDN1A*) were excluded from the dataset when stratifying based on *CDKN1A* (or *FBXW7*) mutation status. The observed similarity was retained in this comparison, emphasizing the presence of shared signaling events that cannot be explained by confounding co-occurring mutations. A detailed look into the maximal common subgraph of CDKN1A and FBXW7 networks revealed a set of genes that have roles in regulating cell cycle progression and proliferation. Figure 3c shows the relevant portion of the subgraph, especially highlighting three genes: *CEBPA*, *CEBPB*, and *CEBPD*, which have roles in cell cycle regulation across different tissues^37,38^ and have been shown to interact with *CDKN1A*^39^. Canonical roles of *CDKN1A* and *FBXW7* further reinforce these networks’ connections to cell cycle regulation. *CDKN1A* encodes for a critical regulator of cell cycle, which has inhibitory activities on CDK – cyclin complexes, including the CDK2 – Cyclin E complex^40,41^. Cyclin E levels can also be regulated by *FBXW7*, through its role in providing substrate recognition for SCF ubiquitin ligases^42,43^. These established roles of *CDKN1A* and *FBXW7* show that regulation of CDK2 – Cyclin E complex activity and in turn, regulation of cell cycle is a common downstream target of their mutations. The pathway view of genes frequently altered in bladder carcinoma generated by TCGA^1^ coheres with this observation, as both *CDKN1A* and *FBXW7* are listed as negative regulators of *CCNE1* and cell cycle progression. Combined together, the dysregulation of molecular events that control cell cycle progression emerges to be one of the common signaling events mutant *FBXW7* and *CDKN1A* converge on.

#### Networks associated with genes recurrently mutated in lung adenocarcinoma

**Figure 4:**
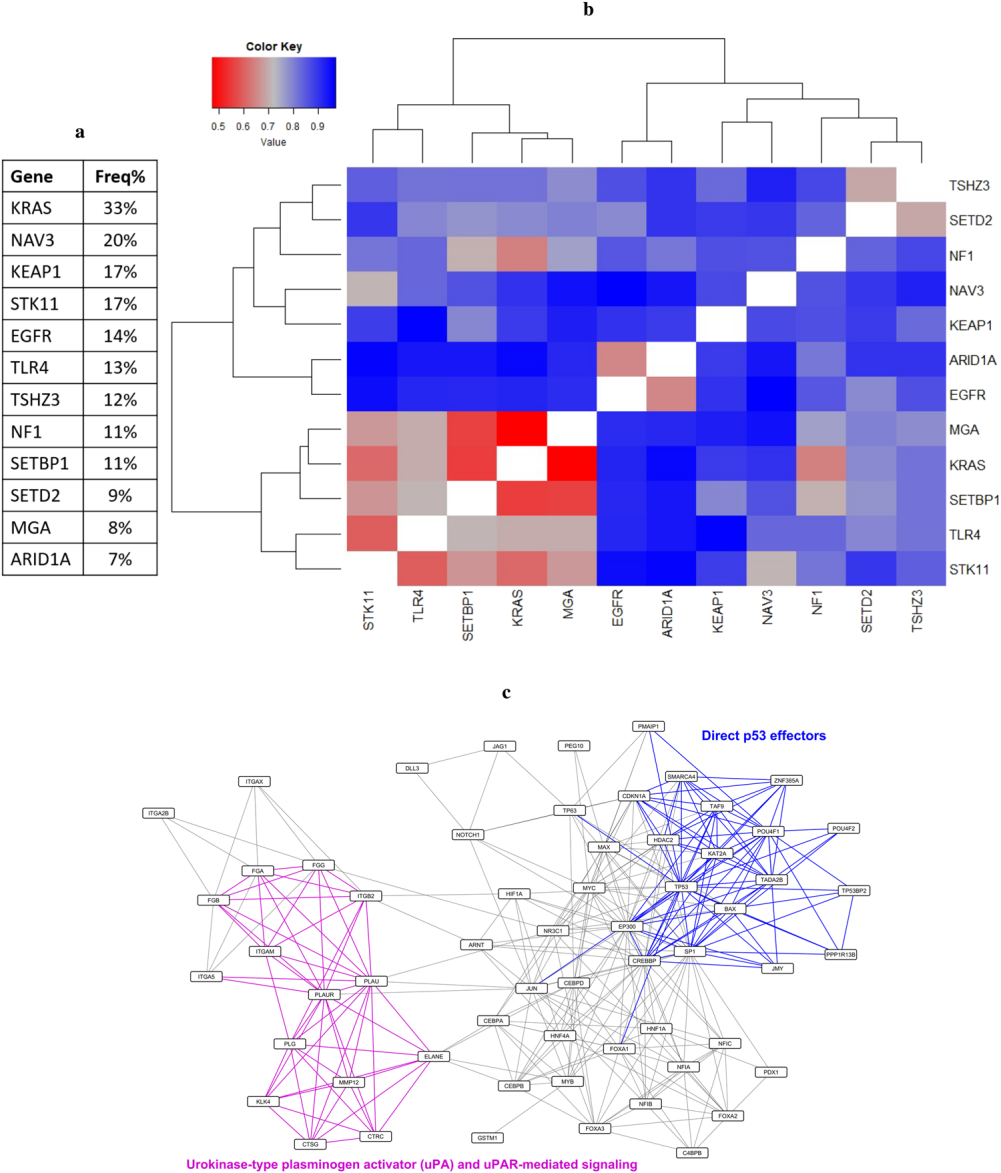
Signaling networks associated with frequently mutated genes in lung adenocarcinoma. (**a**) List of genes included in this analysis and their mutation frequency in the TCGA population. (**b**) Hierarchical clustering of the matrix representing pairwise distances between label propagation-based networks of genes frequently mutated in lung adenocarcinoma. Color code is shown in the upper left corner, where red corresponds to smaller distance values and blue corresponds to higher distance values. (**c**) The maximal common subgraph of TSHZ3- and SETD2-associated networks. Edges belonging to two signaling events listed in the network are highlighted in corresponding colors.

To investigate the molecular events affected by genes recurrently mutated in lung adenocarcinoma patients and the signaling events individual genes potentially converge on, we applied the workflow described above to the lung adenocarcinoma dataset. Genes selected for this analysis based on the literature^2,4,5^ were mutated in at least 7% of patients in the TCGA study (Fig. 4a). For each gene in this list, a corresponding network of signaling changes associated with its mutations was created as described. Then, pairwise distances between each pair of networks were computed to create the pairwise distance matrix of lung adenocarcinoma. The heatmap obtained after performing hierarchical clustering on this matrix is shown in Fig. 4b.

A closer look into this heatmap reveals some patterns that are in line with our current understanding of the functions of certain genes and others that offer additional insights into their functional roles. One case that is a prime example for the former is the similarity observed between networks of KRAS and NF1, two genes whose mutations are mutually exclusive in lung adenocarcinoma patients^2^. Canonical roles of these genes imply that both loss of function mutations in *NF1* and gain of function mutations in *KRAS* can lead to constitutively active MAPK signaling^2,44^, offering a possible explanation for this observed similarity. Another pair of networks that can similarly be expected to have shared signaling changes is KRAS and MGA. Loss of function mutations in *MGA* are proposed as a mechanism for activating MYC signaling^2^ and there are multiple studies that place *KRAS* upstream of MYC signaling^45,46^. Taken together, we can hypothesize that perturbations in MYC activity caused by mutations in *MGA* or *KRAS* might explain the shared signaling changes observed between these two networks. These two pairings provide further support to our idea that studying the relationships between these individual networks reveals functional connections between the corresponding genes and how their mutations can exert shared effects on signaling events, especially highlighting the range of ways a cell can dysregulate an individual pathway.

Following these examples, we turned our attention to pairs of genes whose overlapping biological functions are less intuitive, in order to see whether we can gain more functional information about a given gene or the pathways that are regulated by it by studying its network and similarity patterns. One such example is *TSHZ3*, whose network shares a similarity with SETD2-associated network. With this pairing, it is harder to immediately realize a link between the two proteins and their effects on cell signaling, especially because of our limited knowledge on the role of *TSHZ3* in lung cancer. However, analyzing the maximal common subgraph of the two networks revealed clues about common downstream effects of these genes and a potential insight into *TSHZ3*’s contribution to lung adenocarcinoma biology. Figure 4c shows the maximal common subgraph of TSHZ3 and SETD2-associated networks, highlighting two pathways of interest: “Direct p53 effectors” and “Urokinase-type plasminogen activator (uPA) and uPAR-mediated signaling”. Interestingly, plasminogen activator inhibitor-1 (PAI-1), which is the inhibitor of urokinase-type plasminogen activator (uPA), is a known target of p53^47,48^, and studies show a cross-talk between p53 and plasminogen activator signaling^49,50^. The presence of these signaling events in both of these networks implies that transcriptional activity of p53 and its target genes might be affected by mutations in *TSHZ3* and *SETD2*. Supporting this observation, *SETD2* was shown to contribute to the regulation of p53 signaling by enhancing its transcriptional activities^51^ and in a recent study, *TSHZ3* was identified as an inhibitor of p53 activity in lung cancer cell lines^52^. Overall, these observations highlight p53 signaling and plasminogen activator pathways as critical signaling events that are perturbed downstream of mutations in *TSHZ3* and *SETD2*, proposing a role for these mutations in lung adenocarcinoma tumorigenesis. This also demonstrates the value of focusing on the signaling networks and their similarity patterns, and how it can reveal underappreciated functional roles of frequently altered genes and ways they contribute to tumor biology.

#### Networks associated with genes recurrently mutated in endometrial carcinoma

**Figure 5:**
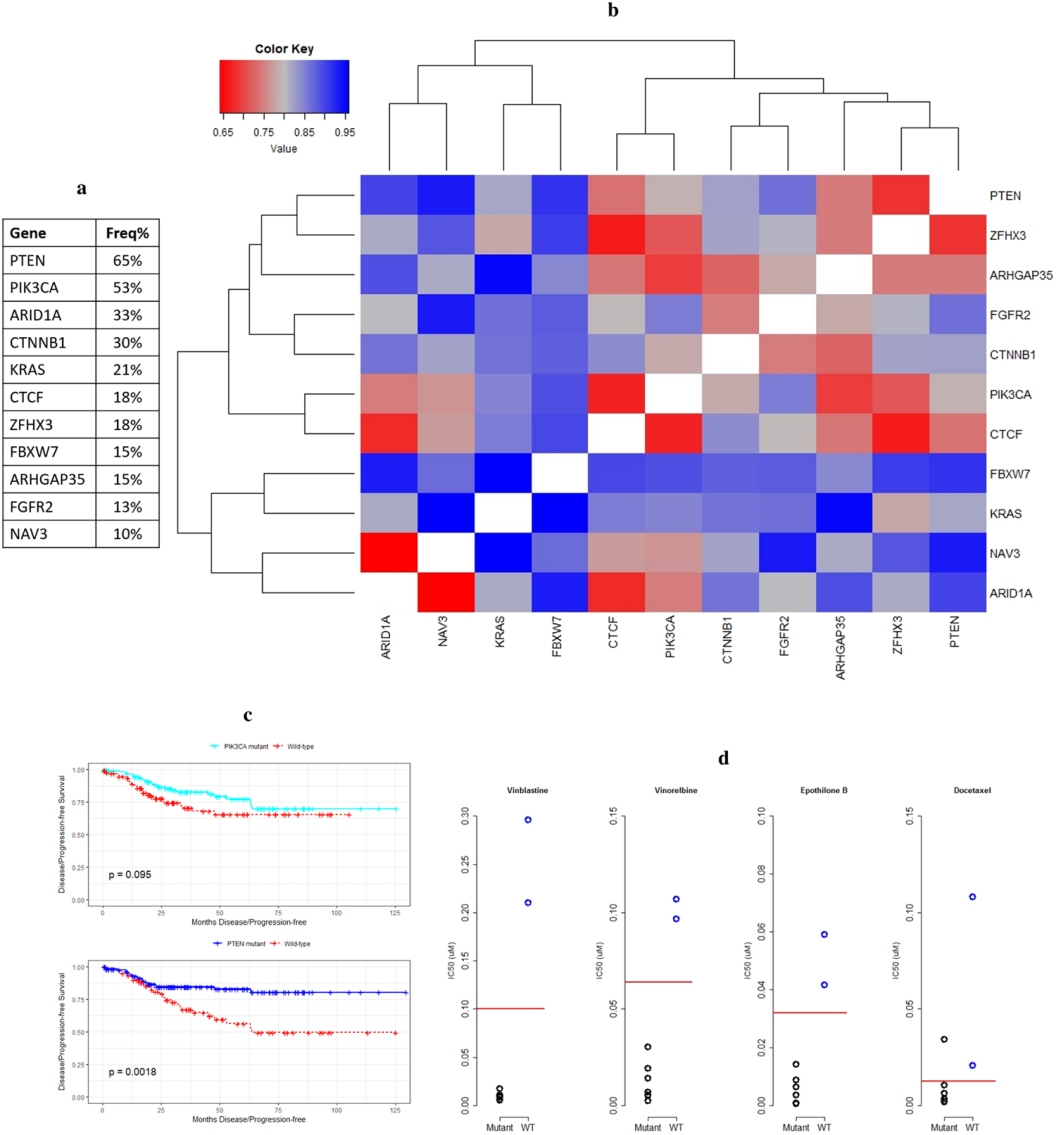
Signaling networks associated with frequently mutated genes in endometrial carcinoma. (**a**) List of genes included in this analysis and their mutation frequency in the TCGA population. (**b**) Hierarchical clustering of the matrix representing pairwise distances between label propagation-based networks of genes frequently mutated in endometrial carcinoma. Color code is shown in the upper left corner, where red corresponds to smaller distance values and blue corresponds to higher distance values. (**c**) Progression-free survival curves of endometrial carcinoma patients are shown for patients with *PIK3CA* mutations in comparison to wild-type patients (top panel) and for patients with *PTEN* mutations in comparison to wild-type patients (bottom panel), along with corresponding log-rank test p-values. (**d**) Each graph depicts IC_50_ values of a microtubule inhibitor, whose name is listed at the top of the graph, measured across a set of endometrial carcinoma cell lines. Black circles represent cell lines that have mutations in either *CTCF* or *ZFHX3* whereas blue circles represents cell lines that are wild-types for both. Red lines represent the maximum screening concentration of each drug, separating cell lines into responder and non-responder categories.

Next, we focused on endometrial carcinoma and the set of genes that are mutated in at least 10% of patients in the TCGA study (Fig. 5a), which are selected based on the literature^3–5^. The same analysis workflow was used to create individual networks of signaling changes for each gene given in this list. Following that, pairwise distances between these networks were computed and the resulting heatmap when this pairwise distance matrix was clustered is shown in Fig. 5b.

Loss-of-function mutations in *PTEN* and gain-of-function mutations in *PIK3CA* are both expected to lead to the activation of PI3-kinase signaling pathway^53^. In our analysis, we observed some overlap between these two networks, in line with this expectation. However, there were also signaling events that are uniquely observed in PTEN and PIK3CA-associated networks, which is an observation supported by studies that examine non-overlapping roles of these two genes^53,54^. Differences observed between progression-free survival trends of these two groups further emphasize underlying biological differences between alterations in *PTEN* and *PIK3CA*. As seen in Fig. 5c, mutations in *PIK3CA* do not lead to a significant deviation in progression-free survival compared to wild-type samples, whereas patients with *PTEN* mutations show significantly improved progression-free survival in comparison to wild-type samples. Overall, these observations hint at the nonredundant roles of these genes beyond the activation of PI3K signaling and emphasize the value of additional knowledge that can be gained on cancer genes by taking a broader look into their putative effects through these networks. Additionally, there are several other pairs of genes with higher levels of similarity, such as networks associated with ARID1A and NAV3, ARID1A and CTCF, or PIK3CA and CTCF. Future work on these pairings has the potential to uncover unexpected connections between their putative downstream effects and novel insight into their contributions to endometrial carcinoma.

#### Identifying druggable vulnerabilities common to genes with similar networks

Through pairwise similarity matrices, we were able to identify networks with high-level similarity across three different tissue types. Our endometrial carcinoma analysis offered especially interesting results as it returned numerous pairs of networks with similarity, where most of the corresponding genes in these pairs - such as *CTCF*, *NAV3*, and *ZFHX3* - are not typically implicated as markers of drug response. This prompted us to take a further look into these pairs to see if they reveal potential druggable vulnerabilities. To connect patterns observed in our distance matrices to drug response profiles, we can hypothesize that alterations in distinct genes linked to similar changes in the signaling network of a given tumor type have the potential to lead to similar changes in drug response profiles, based on the possibility that certain druggable vulnerabilities might arise from specific changes in the signaling network of a cell.

To test this hypothesis, we turned our attention to the Genomics of Drug Sensitivity in Cancer (GDSC) dataset, which offers baseline sensitivities of 1001 cell lines to 265 drugs^55^. We focused on pairs of genes from our endometrial carcinoma analysis with similar networks, with the aim of searching for druggable vulnerabilities that are shared by two genes with similar networks. Based on the sequencing information available on GDSC cell lines, we stratified nine available endometrial carcinoma cell lines into two groups: the ones that have mutations in either gene in the pair and the ones that are wild-type for both. The response profile of each drug was also used to categorize these cell lines into responder and non-responder groups and subsequently, we searched for drugs where mutant and wild-type groups showed patterns of differential response and were separated into opposing response categories. Screening across the available drugs and selected list of pairs of genes revealed an interesting pattern of response to microtubule inhibitors when cell lines were stratified based on mutations in *CTCF* and *ZFHX3*. There were four drugs where only the mutant cell lines were responsive and all wild-type lines were in the non-responder group, satisfying our search criterion of genomic events leading to the stratification of mutant and wild-type groups into distinct response patterns. These drugs were vinorelbine, epothilone B, vinblastine, and OSU-03012. Interestingly, first three of these four drugs all target microtubules. Additionally, there is one more microtubule inhibitor in this drug panel, which is docetaxel, and only one of the mutant lines falls into the non-responder category in the case of docetaxel, with the rest of the cell lines behaving similarly to the patterns observed with the other three microtubule inhibitors. Stratification of the cell lines based on their mutation status and the corresponding separation of response to microtubule inhibitors can be seen in Fig. 5d. Even though the sample sizes for the two groups were small, the consistency across drugs targeting the same molecular event makes this pattern stand out. *ZFHX3* is frequently mutated in endometrial carcinoma patients, however there is not a clear understanding of the functional implications of these mutations in the context of endometrial cancer and this analysis nominates a potential actionable connection between *ZFHX3*, *CTCF*, and regulation of microtubule dynamics. Overall, this example underscores the potential of surveying dysregulated signaling events shared across distinct genomic alterations to find druggable vulnerabilities of tumors.

## Discussion

Mutations in genes that are critical in maintaining cellular homeostasis are among the main events that contribute to tumor development and progression. Understanding the functional contributions of genes to the overall cellular signaling network helps us discover the molecular events that are perturbed when they are mutated and how these perturbations contribute to tumorigenesis. For instance, extensive work on the molecular events downstream of the ErbB family receptors paved the way to our understanding of how mutations in these genes are used by tumors to drive growth and survival^56^. Additionally, discovering functional relationships and cross-talk between different genes revealed that tumors may perturb common signaling events through a multitude of mechanisms; for example, mutations in numerous different genes can lead to the activation of MAPK signaling^57^. These are especially valuable findings, as redundant ways of achieving the same signaling changes have the potential to affect outcomes of therapeutic interventions^58^.

These critical insights gained from individually studying effects of recurrent mutations have motivated us to perform the analysis workflow described above on a variety of genes frequently mutated across different tumor types, in order to gain a perspective on the breadth of changes observed across different tumors. We were especially interested in approaching this problem from a signaling network based point of view, rather than simply focusing on lists of differentially expressed genes. Genes which display dysregulated expression following alterations in a given gene offer us a snippet of the signaling changes that occur when a cell is adapting to these alterations. The label propagation based methodology described in this study expands this limited look into a more cohesive network, highlighting individual signaling or regulatory events that these differentially expressed genes take part in. This in turn can be used to study the range of molecular events associated with mutations occurring in a particular gene, revealing both known and novel downstream effects that are induced by the alterations. Additionally, it is important to keep in mind the possibility that the signaling changes identified based on comparisons between mutant and wild-type samples might not be a direct result of these mutations. They can also be caused by other biological phenomena, where the stratification pattern of samples based on a mutant gene is correlated with this underlying phenotype driving the observed transcriptional changes. So in this instance, these recurrently mutated genes could be seen as markers linked to these signaling changes rather than their cause, revealing a different but still interesting biological insight. Broadly, we can interpret this workflow as building associations and connections between genomic alterations and changes in gene expression patterns and signaling events, creating a bridge between genotype of these tumors to their corresponding phenotypic effects. This offers a different but complementary perspective compared to other applications of label propagation in cancer genomics, such as HotNet^15,16^ and network-based stratification^17^, which highlight subnetworks that are enriched for genes frequently mutated across patients whereas the workflow described here explores the molecular events with dysregulated expression linked to mutations observed in a single gene.

Another key observation made possible by this network-based approach was the range of similarities and differences observed across different genomic alterations. Studying pairs of networks and their similarities revealed signaling events tumor cells converge on through mutations in distinct, and sometimes seemingly unrelated, genes. By computing a distance metric that focuses on shared signaling events, we were able to discover pairs of genes whose mutations lead to the dysregulation of overlapping molecular events. For instance, the similarity observed between CDKN1A and FBXW7 networks underscores the possibility that cell cycle dysregulation in bladder carcinoma can be achieved by directly inactivating a CDK inhibitor or alternatively, by inactivating a regulatory protein. Additional key insight gained through this approach includes identifying potential roles for genes whose mechanistic connections to tumorigenesis are unclear. For example, the contribution of p53 and its downstream signaling events, such as plasminogen activator signaling, to the maximal common subgraph of the TSHZ3 and SETD2 networks offers further support to the hypothesis that *TSHZ3*’s role in lung adenocarcinoma includes regulation of p53 activity^52^. On the other hand, the PTEN and PIK3CA networks generated through our analysis of endometrial carcinoma datasets highlight the underappreciated divergence in signaling changes that may occur as a result of mutations in these genes. In most cases, effects of their mutations are linked to the activation of PI3-kinase pathway. In line with the growing evidence that these mutations might have distinct roles apart from PI3-kinase pathway activation^54^, our analysis points to additional, non-overlapping signaling changes that are associated with them. Overall, comparing and contrasting of dysregulated signaling networks of different genes offered us a unique look into the intricate molecular changes tumors rely on to survive, emphasizing the range of known and unexpected connections present across genes.

Lastly, we showed how these patterns of similarity can be used to search for novel therapeutic connections. Similarities reflected in molecular states induced by two mutated genes also have the potential to be linked to similar drug response patterns. This means that we can use genes connected to similar downstream signaling changes to define a new search space that stratifies samples based on the combination of these genes rather than simply stratifying based on individual genes. This type of stratification scheme has the potential to uncover unique relationships between a set of genomic markers and drug response, that could otherwise be missed by traditional “one mutation/one drug” correlation analyses that are commonly used to search for markers of response^55^. Additionally, this offers a new approach for searching higher-order interactions between genomic markers. Identifying combinations of markers can become computationally challenging as the combinatorial search space quickly grows with the number of individual markers considered. By only searching within gene pairs that already share a biological connection, we are both focusing on a smaller search space and prioritizing pairs of alterations that might have higher likelihood of leading to similar drug response patterns. For instance, by stratifying endometrial carcinoma cell lines based on mutations in both *CTCF* and *ZFHX3*, we observed a pattern of differential response to microtubule inhibitors, and this pattern would not have been apparent if we stratified cell lines based on mutations in individual genes. Therefore, this detailed look into the dysregulated state of signaling networks has the potential to inform discoveries of unique markers of drug sensitivity.

One way this workflow can be further improved upon is by replacing the input network with other types of networks to explore interactions representing different biological perspectives that are not covered by PID. For instance, if one is interested in studying the range of potential physical interactions between the elements of the gene set, a broader network built experimentally to explore previously uncharacterized interactions, such as the human interactome network generated by Rolland *et al*.^59^, can be used as the input network. Alternatively, a more specialized network that is tailored to genes expressed in specific tissues or cell types can be used to offer a more detailed insight by focusing only on molecular processes that are active in that particular biological setting.

The approach presented here may also pave the way to a variety of follow-up studies. For instance, a focused look into the network of a gene whose functional roles are poorly understood can be performed to detail the key mechanisms by which it contributes to tumor progression and development. This can be followed with experimental validation of these observations to characterize the functional contributions of signaling events observed in the network. Similarly, for pairs of similar networks lacking an evident link explaining the observed overlap, experimental studies can be performed to discover links between these individual genes and pathways they converge on. The unique search space of druggable vulnerabilities generated by focusing on genes with similar networks can be exploited further by expanding this study to additional tumor types and genes. Additionally, the ability to connect genomic alterations to gene expression alterations can be used to annotate and characterize expression changes that are induced by mutations in the non-coding region of the genome and improve our understanding of how these often unexplored alterations contribute to tumorigenesis. It is worth mentioning that this workflow can be used to focus on any gene set of interest, opening up the potential to generate networks that offer an in-depth analysis of a variety of phenotypes. Collectively, this study emphasizes the potential of the label propagation-based approach to expand a set of genes to create a more cohesive network view that reveals the underlying signaling connections between the elements of the gene set.

## Methods

### Label propagation-based workflow

Zhu *et al*. developed a graph-based label propagation algorithm for solving semi-supervised learning problems using harmonic functions^60^. In an instance of graph based semi-supervised learning, there is a graph *G*, which in total has *n* nodes and *e* edges. These *n* nodes belong into two groups: there are *u* unlabeled nodes and *l* labeled nodes - each with its own label value *y*_*i*_. The edge set *e* is represented with a symmetric weight matrix *W*, where *w*_*ij*_ is nonzero if there is an edge between nodes *i* and *j*. We also define a function *f*. For labeled nodes, this function has the value of their labels *y*_*i*_. The solution to the learning problem is calculating the value of this function *f* for each unlabeled node. These values can then be used to classify unlabeled nodes into distinct classes represented by labeled nodes, typically by picking a threshold value for *f* and separating unlabeled nodes into classes accordingly by comparing their *f* value with the threshold. The following iterative algorithm can be used to perform these calculations:

$$\begin{array}{l}{\rm{Set}}\,f({x}_{i})={y}_{i},\forall i=1\ldots l\\ {\rm{Randomly}}\,{\rm{initialize}}\,f({x}_{j}),\forall j=l+1\ldots l+u\\ {\rm{For}}\,t=1,\ldots ,T\,{\rm{and}}\,j=l+1,\ldots ,l+u\\ \,\,f({x}_{j})=\frac{{\sum }_{k=1}^{l+u}\,{w}_{jk}\,f({x}_{k})}{{\sum }_{k=1}^{l+u}\,{w}_{jk}}\end{array}$$

This is the generic description of the label propagation algorithm that we used in our approach. In our context, the graph will be a biological network, where nodes represent genes and edges represent interactions or functional relationships between them. The gene set of interest will form the labeled node set, which means that there will only be one class of labeled nodes. As a result, interpretation of the resulting *f* values will be different than the generic case described above. Rather than picking one threshold value and comparing each unlabeled node’s value against it, we will assess whether an unlabeled node’s *f* value is significantly higher than a value that can be obtained by chance alone. This way we will be focusing on identifying unlabeled nodes which receive a significant amount of diffusion from labeled nodes. To achieve this, we first generated a set of random networks with the same degree distribution as the original network, by using a network randomization algorithm based on the “edge switching” principle described by Maslov and Sneppen^61^. After creating random networks, the label propagation algorithm was run with the exact settings as the original network on each of these random networks individually. p-values for each individual unlabeled node’s *f* value were computed by comparing the values obtained with the random networks with the value obtained with the original network. Then, these p-values can be used to determine which unlabeled nodes have significantly high *f* values. Benjamini-Hochberg correction^62^ was applied to offer a more conservative control on false discovery rate.

As mentioned above, we also need to specify an input network. There are a variety of options that can be used, each with a different focus. Networks that focus on pairwise physical interactions between proteins that are obtained via high-throughput experiments, such as yeast two-hybrid, would be useful if one is interested in high coverage of potential interactions. Alternatively, manually curated pathway based networks can be used if the focus is more on functional interactions between proteins in the context of signaling and regulatory pathways. We are more interested in the latter - we would like to connect functionally related genes to each other and place them into their respective positions in signaling pathways rather than simply identify if there are interactions between them. Additionally, experimentally generated physical interaction networks may contain interactions that are not physiologically relevant or have higher rates of false positive interactions compared to manually curated networks that rely on interactions that have already shown to be biologically relevant. Considering these trade-offs and our aim of placing these genes into the relevant signaling context, we decided to use the Pathway Interaction Database (PID)^18^, which is a high-quality resource curated by experts that represents a variety of currently known signaling and regulatory pathways^63^. To obtain PID pathway data, we used Pathway Commons (PC) database^64^. PC preserves pathway membership annotation of this network while presenting it in a pairwise interaction format, where each pair of interacting genes is listed alongside the pathway(s) that this particular interaction takes part in. As a result, for each given edge in the subnetwork identified through label propagation, we have information on the corresponding pathway(s) that it belongs to. This mapping was used to determine the biological pathways that collectively amount to the subnetwork obtained at the end of label propagation, providing us with a list of signaling events that the starting genes are a part of or interact with. Within this subset of pathways identified, we can additionally emphasize the ones with more substantial contribution to the interactions represented in the network and search for pathways that are enriched in this network by performing hypergeometric tests. Signaling events highlighted in subnetworks shown in figures above were in the group of events that were significantly enriched in their respective networks.

Overall, the workflow is as follows. PID data was converted into a weight matrix *W* to be used as the input network. A set of genes of interest, such as a gene expression signature, formed the set of labeled nodes. Every gene is represented by a unique node in the PID network so the genes of interest was mapped to their respective nodes to obtain the set of labeled nodes. To initialize *f* function’s values, labeled nodes were assigned 1 and unlabeled nodes were assigned 0. The iterative algorithm was then run *t* times, which was a value specified by the user. Following this, edge switching algorithm was used to generate a set of 1000 random networks. The label propagation algorithm was then run on each of these random networks separately, with the remaining parameters staying exactly the same. p-values based on these label propagation runs were then calculated to identify the nodes that have *f* values significantly higher than what would be expected by chance. The significance thresholds at this step can be adjusted, based on how stringent one wants results to be. As the final step, the resulting subnetwork’s edges were mapped to the biological pathway(s) that they belong to based on annotations stored in the PID network, to identify the set of signaling events that are represented with a given subnetwork. To further prioritize molecular events with significance, we identified pathways that were significantly enriched in this network.

#### Alternative approaches for subnetwork identification

In the workflow detailed above, label propagation is the method of choice for extracting a relevant subnetwork from a biological network, where the resulting subnetwork represents the local neighborhood of a starting set of nodes. There are additional methodologies that can be used to extract subnetworks based on different properties of a network of interest. Some of these alternative methods focus on connecting a given set of starting nodes to each other through their direct interactors or shortest paths between them^9,10^. However, in comparison to label propagation, these methods traverse a limited subset of the network as they tend to focus on identifying shortest paths. If the network of interest is weighted, heuristics connecting seed nodes to each other through shortest paths between them under the constraint of a scoring function can also be used^65^. With these approaches, high-throughput datasets, such as gene expression, are typically used as the first step to identify sets of genes of interest which then form the foundation of the subnetwork. As a result, the starting set of genes influences the resulting network as the search space is focused on exploring their neighborhood instead of examining global properties of the network.

Alternatively, there are methods that consider the global topology of a given network to identify potential subnetworks of interest, such as module finding algorithms that reveal highly interconnected subnetworks^66^. These modules or clusters are of interest based on the assumption that sets of proteins that are functionally related, such as protein complexes, will be closely connected to each other in a biological network in contrast to a random group of proteins with no functional relationship. These subnetwork identification methods can also be extended to integrate gene expression or other high-throughput datasets with network analysis steps, enabling to explore the connections between changes observed in experimental datasets and topology of a network. One common way of achieving this is assigning weight to edges based on correlation of expression values^67^ or covariation of expression levels^68^ between interaction partners. The next step is then identifying clusters of interconnected nodes within this weighted network with algorithms such as Markov clustering^67^ and simulated annealing^68^, revealing topologically close genes with similar expression patterns. An alternative data integration strategy is offered by Wang *et al*., where nodes are weighted based on their level of association with a phenotype of interest and edges’ weights depend on the strength of a given interaction^69^. The subnetworks of interest extracted from these networks, based on an optimization framework, reveal densely connected set of nodes with strong association to the phenotype.

The methods outlined above offer different perspectives for extracting interesting subnetworks from a given biological network and one point of consideration in determining the appropriate method is the biological problem at hand. Methods searching for highly interconnected subnetworks, either in an unweighted network or in one weighted based on experimental datasets, offer a way to search the whole network for relevant patterns, taking advantage of its global properties. However, they tend to return small subnetworks that represent a focused functional unit, such as a protein complex, a group of disease related proteins, or a specific molecular event, that are typically disconnected from each other. Therefore, we can identify groups of highly connected genes that are acting in concert but the connectivity and crosstalk between them are not immediately apparent. As a result, these would be applicable to cases where we expect to identify multiple distinct functional units that best satisfy the weight and connectivity constraints within the full network. On the other hand, approaches that focus on traversing a network starting from a set of nodes will be more equipped to provide a more connected path across complexes and pathways identified. The main focus of these ideas is bridging the gaps between a given set of nodes by revealing paths connecting them to each other and to neighboring nodes, as a result they tend to return one connected component representing the collective neighborhood of the starting set. The caveat is that some parts of the network will remain unexplored as they are not in the search space defined based on proximity to starting set of nodes. Therefore, the initial step of selecting these nodes is critical to success of identifying biologically relevant subnetworks. Bearing these points in mind, we chose a label propagation based approach, as our aim was to build connected subnetworks that bridge the gaps between a variety of distinct signaling events summarized by a given gene expression signature.

### Characterizing the workflow

**Figure 6:**
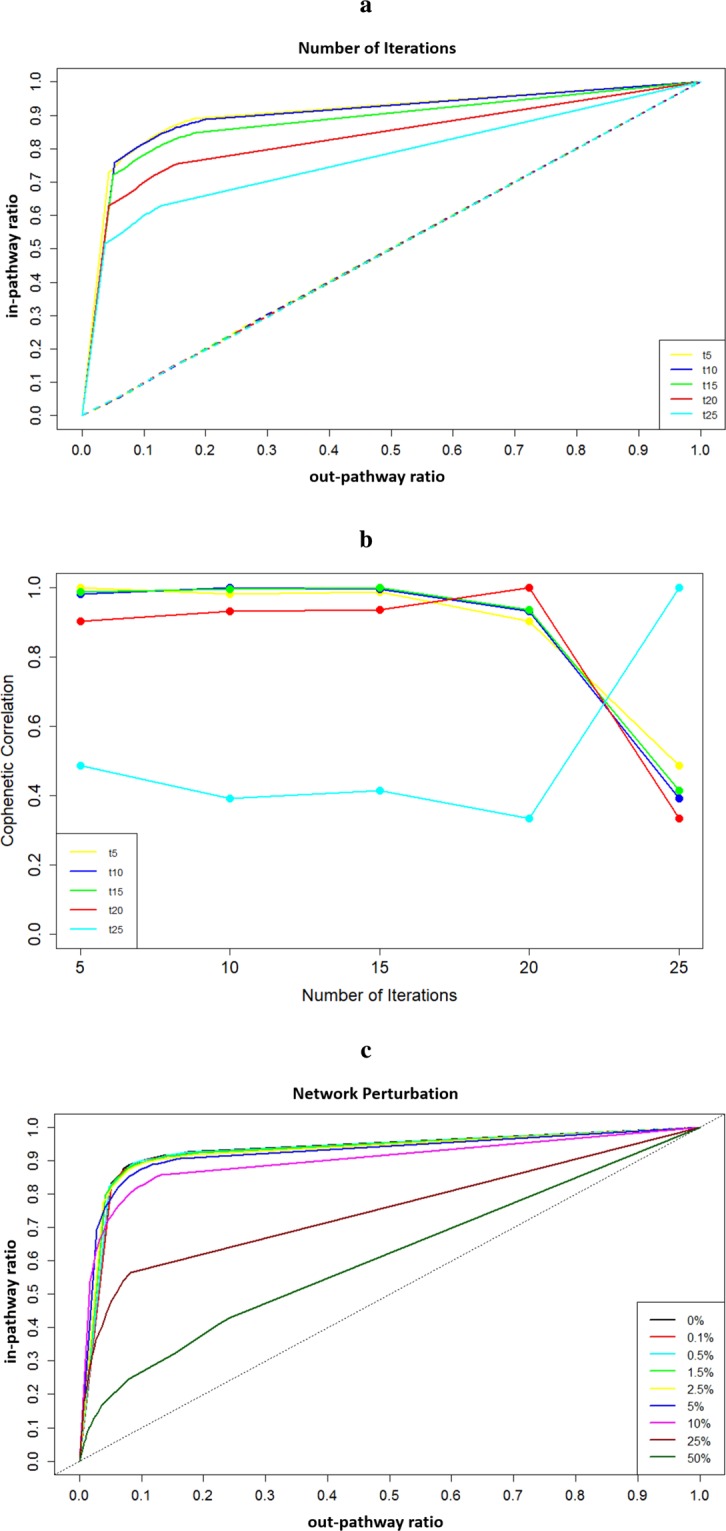
Characterization of the workflow. (**a**) The results of tests performed to identify suitable values for number of iterations parameter. Each line represents average in-pathway and out-pathway ratios obtained with each different value of the number of iterations parameter. Color-key for corresponding number of iteration values is shown in the lower-right corner. (**b**) Cophenetic correlation values observed between clustering structures of pairwise distance matrices obtained across a range of values of number of iterations parameter. Color-key for corresponding number of iteration values is shown in the lower-left corner. (**c**) The results of tests performed to measure the effects of network structure on label propagation performance. Each line represents average in-pathway and out-pathway ratios based on the results obtained with networks with varying levels of perturbation. Color-key for corresponding perturbation percentages is shown in the lower-right corner.

As the number of iterations parameter, *t*, is an important determinant in the results the label propagation returns, we sought to determine a value that will result in a high discovery rate across many different cases. To run tests for determining optimal value of number of iterations parameter, we selected a list of pathways that are represented in the PID network and identified the genes that form these pathways. A subset of genes participating in a given pathway was sampled without replacement to form the set of labeled nodes. Multiple sampling sizes were used for each pathway, resulting in labeled node sets containing 10, 20, 35 or 50 genes and multiple sets were sampled for each size. Multiple starting pathways and differing sizes of initial seed gene sets were used to ensure that the value is not optimized based on a single pathway or starting gene set size value, but rather that it reflects a suitable value that will perform well across a range of inputs and settings. Following this random sampling to generate sets of seed genes, label propagation algorithm was run as described above. Number of iterations parameter was changed each time, and total range of values used were *t* = 5, 10, 15, 20, and 25. At the end of an individual run, recovery ratio of genes belonging to the pathway of interest were computed. “in-pathway” recovery rate corresponds to the ratio of number of significant genes that belong to the pathway of interest over the total number of genes that form the pathway, excluding the genes that are part of the seed set. This number represents how many additional genes that are part of the starting pathway of interest we have been able to identify as significant through label propagation. “out-pathway” recovery rate is the ratio of number of remaining significant genes that are not part of the pathway of interest to the total number of genes in the complete PID network that are not part of the pathway of interest. This number represents additional neighboring genes that are identified to be significant, which belong to different pathways. These in-pathway and out-pathway recovery rates were computed at the end of every single run, across a range of significance thresholds. Then, average values were obtained for each different value of the number of iterations parameter by computing the mean of in-pathway and out-pathway ratios obtained with a given *t* value across all different pathways and starting gene set sizes. As it can be seen in Fig. 6a, *t* = 5 and *t* = 10 were the best performers in maximizing in-pathway recovery rate, with *t* = 10 returning slightly higher in-pathway rates under some conditions. As a result, in all upcoming runs, we used 10 as the value of the number of iterations parameter.

We were also interested in studying the robustness of results to parameter selection. To test this, we focused on the relationship between one of the tissue types we studied, namely bladder carcinoma, and number of iterations parameter. The analysis performed with each individual gene in the bladder carcinoma list was repeated with different values of number of iterations, including *t* = 5, 10, 15, 20, and 25. Resulting networks were grouped based on the value of number of iterations used and within each of these five groups, we computed the pairwise distances between the ten networks corresponding to individual genes to generate pairwise distance matrices. To quantify the level of difference between these five distance matrices and assess the extent of stability found in the clustering of networks based on their similarity, we computed the cophenetic correlation between each individual matrix’s clustering. The resulting pattern is shown in Fig. 6b, implying consistent clustering of resulting networks across a range of number of iterations values. Distances obtained with *t* = 5, 10, and 15 show almost perfect correlation with each other, which shows a level of robustness to parameter selection. The trend across the full range of values tested is also in line with trends seen in Fig. 6a. As seen in Fig. 6a, the in-pathway recovery rate starts to decrease at *t* = 20 with a further decline at *t* = 25. Similarly, Fig. 6b shows that we observe a slight decrease in cophenetic correlation at *t* = 20 and smallest correlation is observed with *t* = 25. Taken together, Fig. 6a,b can be used to identify a range of parameters that returns a robust and high in-pathway recovery rate.

A secondary round of analysis, shown in Fig. 6c, focused on understanding the robustness of this approach with respect to perturbations in the network structure. Specifically, we sought to determine how dependent the results are on the particular topology of the underlying network, which may vary according to human annotation. In a large network like PID, we reasoned that changing the connectivity of a very small percentage of edges should create only minor differences in the complete topology of the network. This implies that if we run label propagation on this minimally perturbed network, we would expect to see results that are substantially similar to the results obtained with the unperturbed network. Failure to observe this correspondence would suggest that the subnetworks obtained are not robust to small perturbations and this would decrease our confidence in the validity of these subnetworks representing a statistically significant neighborhood. To test this concept, we created a set of networks where a percentage of the PID network’s edges were rewired. To create networks with different levels of perturbation, edge switching algorithm was used with a slight variation^61^. Normally, the number of switching steps performed is a multiple of the total number of edges to ensure the edges are properly mixed. However, in this case, a limited number of switches were performed to ensure that only a subset of edges’ connectivity changes, resulting in a network where only a portion of the network is different from the original one. We wanted to perform tests across a range of networks with increasing levels of perturbation, so the number of switches performed was adjusted accordingly to achieve the desired level of perturbation to the overall topology of the network. At the end, the percentage of rewired edges were 0.1%, 0.5%, 1.5%, 2.5%, 5%, 10%, 25%, and 50% of the total number of edges in the complete PID network. Additionally, multiple perturbed networks were created at each of these levels. For this test, subsets of genes participating in a pathway from PID were sampled without replacement to obtain multiple different sets of labeled nodes. These labeled nodes and perturbed networks were then used as inputs to the label propagation algorithm, where the number of iterations parameter was set at *t* = 10, generating an individual subnetwork for each combination of labeled node set and perturbed network. Ratios of in-pathway and out-pathway recovery were computed as described above. Results obtained with networks with same perturbation level were averaged to obtain an individual curve for each perturbation level. Figure 6c shows the in-pathway and out-pathway ratios we obtained at different perturbation levels. The networks with very small perturbation – 0.1% and 0.5% – have curves that are almost the same as the original network, with 1.5% and 2.5% following very closely. This implies that introducing small perturbations to the network did not lead to the complete loss of performance in identifying in-pathway genes. After that point, increasing perturbations led to decreased success in identifying genes belonging to the pathway of interest as the network becomes more and more divergent from the original network. This pattern matches our expectations, which in turn implies that the subnetworks we obtain are robust communities that are connected together through meaningful traversal of the graph and are not spurious subsamplings of the complete network.

### Computing distance between networks

**Figure 7:**
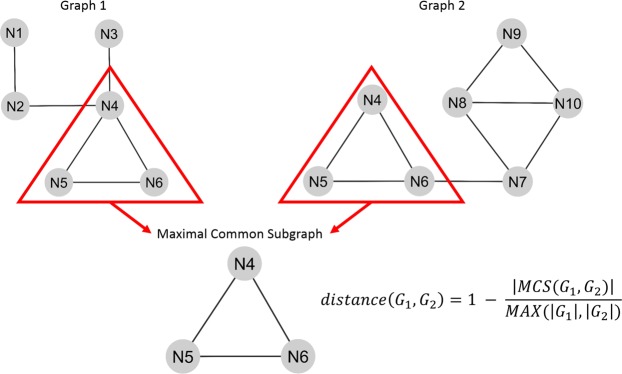
Description of the distance metric. Two graphs are shown to visualize the search space and highlight the corresponding maximal common subgraph.

In order to base comparisons between multiple networks on a well-defined quantitative measure, we employed a distance metric that is based on the concept of a maximal common subgraph^19^. This metric, described visually in Fig. 7, relies on identifying the maximal common subgraph of two given graphs. When searching for common subgraphs between two graphs, we constrained the search space to the nodes sharing the same label. We imposed this constraint because of the nature of biological networks and information represented by node labels. A biological network’s topology represents our knowledge of how genes interact with each other. However, it is not the only source of information in these networks - the labels of nodes are an additional source of information as they represent the genes in a non-redundant fashion. Therefore, when comparing biological networks to identify similarities, it would be misleading to ignore node labels and base the similarity search solely on the connectivity of edges. This might lead to identification of subgraphs with exactly same connectivity but containing different genes and from a signaling perspective, this would represent functionally different subnetworks. Therefore, we instead search for the nodes that are connected in the same manner and that also have the same labels, to make sure that isomorphic subgraphs we identify represent biologically meaningful common subnetworks. Using this constrained search space, we search for a graph *G*_MCS_, where both graphs *G*_1_ and *G*_2_ have a subgraph isomorphic to *G*_MCS_. Of all possible subgraphs that satisfy this criterion, the one with the biggest node size will be the “maximal common subgraph”. Following identification of this subgraph, the distance between two graphs can be computed based on the following formula:

$$d({G}_{1},{G}_{2})=1-\frac{|{\rm{MCS}}({G}_{1},{G}_{2})|}{{\rm{\max }}(|{G}_{1}|,|{G}_{2}|)}$$

To test the performance of this distance metric and to assess how well it performs in quantitatively reflecting shared signaling events, we ran a set of tests with pathways found in the PID network. We randomly selected five genes from three separate pathways and combined them to form the seed gene sets. “Noncanonical Wnt signaling pathway”, “E2F transcription factor network” and “Regulation of nuclear SMAD2/3 signaling” were the three pathways initially selected for this step. Two separate seed gene sets were created by randomly selecting genes from these three pathways. With each individual set, label propagation was run and then, the distance between these two networks were computed based on the metric described above. This test compares two presumably similar networks, both containing genes belonging to the same three pathways and therefore providing a range of expected distance values that represent similar networks. Following this, we ran additional tests with the aim of creating networks with decreasing levels of similarity to the initial network in order to test if these would correspond to increasing distance values. We generated three sets of seed genes where one of the original three pathways was replaced with another pathway and three sets of seed genes where two of the original three pathways were replaced with other pathways. Finally, to simulate a case with completely different networks, we created two sets of seed genes where the three pathways were completely different from the original three. Particular combinations of pathways and the distance values obtained when these networks were compared to the first network are shown in Supplementary Table S2. As it can be seen, the distance values are increasing with decreasing level of overlap in the initial set of sampled pathways. The smallest distance is observed when the same set of pathways are used to obtain seed genes. This is followed by cases where two out of three networks stay the same and then by cases where only one out of three networks stays the same. As anticipated, the highest distance values correspond to the cases where seed genes were obtained from completely different pathways. This supports the idea that in cases where we would not expect similarity, the distance metric returns values in agreement with those expectations. Therefore, we relied on this metric to define the levels of similarity observed across different networks when we were searching for convergent signaling events shared across networks.

### Analysis of TCGA datasets

RNA-seq gene expression data and mutation data from urothelial bladder carcinoma, lung adenocarcinoma, and uterine corpus endometrial carcinoma patients were obtained through TCGA data portal. For each given tissue, genes of interest were selected based on frequently mutated genes highlighted in their respective TCGA publications, TumorPortal^5^, and Kandoth *et al*.’s study across tumor types^4^. Quality control requirements of TCGA, such as excluding samples with low tumor purity and validating identified somatic mutations through targeted resequencing and based on RNA-seq datasets, along with variant allele fraction (VAF) distributions examined by Kandoth *et al*.^4^ revealing that these mutations do not have low VAFs increase our confidence in the fact that the mutations we focused on were harbored in these samples at a relevant level. For each gene in a given tissue’s frequently altered list, we generated a gene expression signature that reflects genes whose transcriptional state is altered in the presence of mutations occurring in the gene of interest. For this step, mutation datasets were used to stratify patients into mutant and wild-type groups: samples with any non-silent mutation were categorized into the mutant group and remaining samples without any mutation in the gene of interest formed the wild-type group. Then, gene expression profiles of these two groups were compared to identify genes that were differentially expressed, via Bayesian approximate kernel regression (BAKR) model^70,71^. This process was performed for each gene individually, in the end to obtain a set of gene expression signatures for each tissue type. These signatures were then used to identify the labeled node set for label propagation approach, as detailed above. The number of labeled nodes used for each individual run ranged from 25 to 60 across the different genes tested. The number of significant nodes identified through propagation did not strongly depend on the size of the seed gene set, as the weak correlation between the two can be seen in Supplementary Fig. S2. After running label propagation with each signature individually, we obtained a subnetwork of the PID network, representing the signaling and regulatory events that are perturbed. Finally, within each tissue type, distances between all pairs of genes’ networks were computed based on maximal common subgraph based metric described above to generate pairwise distance matrices.

### Analysis of drug response

Genomics of Drug Sensitivity in Cancer (GDSC)^55^ project’s web portal (www.cancerrxgene.org) was used to obtain drug response profiles and mutation status of screened cell lines. Each drug in this panel was tested across a range of concentrations to create a dose response curve in each cell line and these curves were then used to calculate IC_50_ values of each drug. For a given drug, we used its maximum screening concentration as a decision boundary to separate the screened cell lines into two classes: cell lines with IC_50_ values greater than maximum screening concentration of a given drug were deemed non-responsive and remaining cell lines with an IC_50_ value smaller than the maximum screening concentration were categorized into the responsive class. Additionally, we identified nine cell lines that were categorized as derived from uterine corpus endometrial carcinoma by GDSC and listed pairs of genes with similar networks from endometrial carcinoma analysis, as shown in Fig. 5b. For each pair of genes with similar networks, we stratified these nine cell lines into two groups based on the mutation dataset: the ones that have a mutation in either gene in the pair formed the mutant group and the ones that do not have any mutations in both genes formed the wild-type group. Our aim was to search for pairs of mutations whose presence led to a separation from the wild-type group in drug response pattern and categorization of mutant cell lines into the opposite response class compared to the wild-type cell lines. For instance, for a given gene pair A and B, we searched for drugs where all responder cell lines had mutations in either A or B and all non-responder cell lines were wild-type for both (or vice versa). To identify these drugs, we analyzed the stratification of mutant and wild-type groups with respect to the boundary defined by the maximum screening concentration and for each gene pairing, we computed the ratio of mutant and wild-type groups in responder and non-responder classes. Based on these ratios, we identified drugs where all mutant cell lines were in the responder class (or non-responder) and all wild-type cell lines were in the non-responder class (or responder), leading to the stratification of mutant and wild-type groups into non-overlapping categories.

### Network visualization

Cytoscape^72^ was used to generate all network views.

## Supplementary information

SupplementaryInfo

## Data Availability

The Cancer Genome Atlas data referenced during the study are available in a public repository from the https://cancergenome.nih.gov website. The Genomics of Drug Sensitivity data referenced during the study are available in a public repository from the http://www.cancerrxgene.org website. The Pathway Interaction Database (PID) network referenced during the study is available in a public repository from the http://www.pathwaycommons.org website.
