# Supplementary material for: Label propagation defines signaling networks associated with recurrently mutated cancer genes
Source: Sci Rep. 2019 Jun 28;9:9401. doi: 10.1038/s41598-019-45603-3 (PMC6599034; doi:10.1038/s41598-019-45603-3)
Supplement: Supplementary file 1 — SupplementaryInfo [file 41598_2019_45603_MOESM1_ESM.pdf]

Supplementary Information

Label propagation defines signaling networks associated with recurrently mutated cancer genes

Merve Cakir [1,2], Sayan Mukherjee [3,*], and Kris C. Wood [2,*]

[1] Program in Computational Biology and Bioinformatics, Duke University, Durham, NC, 27708, USA
[2] Department of Pharmacology and Cancer Biology, Duke University, Durham, NC, 27710, USA
[3] Departments of Statistical Science, Mathematics, Computer Science, Biostatistics & Bioinformatics, Duke University, Durham, NC, 27708, USA
[*] sayan@stat.duke.edu; kris.wood@duke.edu

Index	Name	P-value	Adjusted p-value	Z-score	Combined score
1	ErbB1 downstream signaling_Homo sapiens_30d60550-6192-11e5-8ac5-06603eb7f303	4.390e-8	0.000006015	-1.81	30.70
2	mTOR signaling pathway_Homo sapiens_559dd850-6194-11e5-8ac5-06603eb7f303	0.000007786	0.0005333	-1.64	19.24
3	Nongenotropic Androgen signaling_Homo sapiens_843e2f77-6194-11e5-8ac5-06603eb7f303	0.00005028	0.002296	-1.47	14.58
4	Regulation of Telomerase_Homo sapiens_4dfe97ca-6195-11e5-8ac5-06603eb7f303	0.00009627	0.002473	-1.55	14.35
5	PDGFR-beta signaling pathway_Homo sapiens_c901a3e4-6194-11e5-8ac5-06603eb7f303	0.0002533	0.003852	-1.65	13.65
6	GMCSF-mediated signaling events_Homo sapiens_095aa3ef-6193-11e5-8ac5-06603eb7f303	0.0001047	0.002473	-1.41	12.90
7	Glucocorticoid receptor regulatory network_Homo sapiens_dfba0dfb-6192-11e5-8ac5-06603eb7f303	0.0002506	0.003852	-1.50	12.47
8	IFN-gamma pathway_Homo sapiens_51b1ed75-6193-11e5-8ac5-06603eb7f303	0.0001590	0.003112	-1.26	11.05
9	Fc-epsilon receptor I signaling in mast cells_Homo sapiens_86cd7795-6192-11e5-8ac5-06603eb7f303	0.0006712	0.006268	-1.14	8.33
10	Role of Calcineurin-dependent NFAT signaling in lymphocytes_Homo sapiens_61bdd46d-6195-11e5-8ac5-06603eb7f303	0.0004757	0.005430	-1.08	8.27

49	Validated targets of C-MYC transcriptional activation_Homo sapiens_61d3b115-6196-11e5-8ac5-06603eb7f303	0.1021	0.1749	1.79	-4.07
53	Validated targets of C-MYC transcriptional repression_Homo sapiens_6bbdafa6-6196-11e5-8ac5-06603eb7f303	0.06940	0.1339	1.64	-4.37

58	TCR signaling in naive CD4+ T cells_Homo sapiens_0c2862fa-6196-11e5-8ac5-06603eb7f303	0.3564	0.3643	4.50	-4.64
69	TCR signaling in naive CD8+ T cells_Homo sapiens_15a017bb-6196-11e5-8ac5-06603eb7f303	0.3056	0.3297	4.84	-5.73

Supplementary Figure S1: Signaling pathways enriched in the "glucocorticoid resistance" signature. Enrichr was used to identify signaling pathways annotated by PID that are enriched in this signature. The top panel lists the top 10 pathways, sorted based on the combined score, along with their p-values and z-scores. Middle and bottom panels show the values corresponding to the pathways that were identified in the network obtained with label propagation (shown in Figure 2c), highlighting the lack of significant enrichment in the original signature.

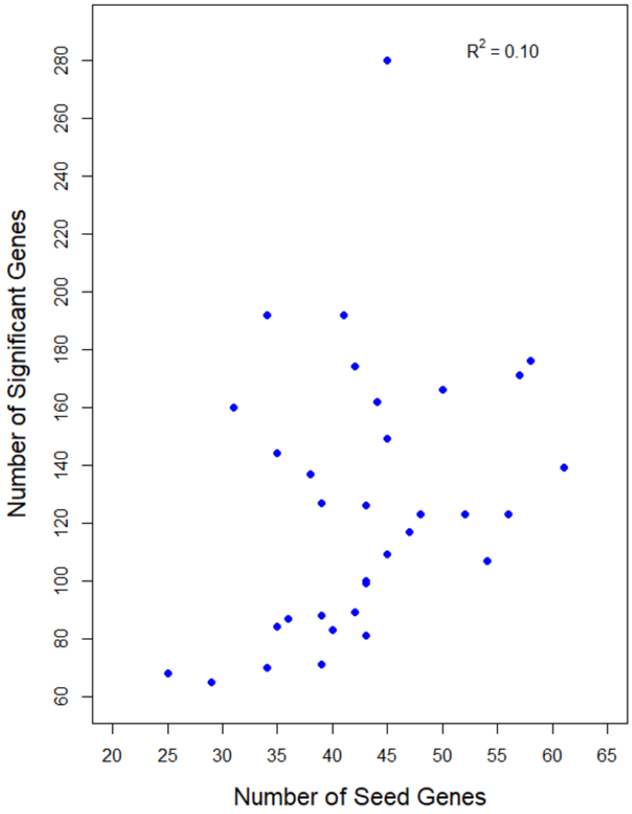

Supplementary Figure S2: Relationship between the number of seed genes and the resulting number of significant genes. The x-axis represents the range of number of seed genes used across TCGA analyses depicted in Figures 3, 4, and 5. The y-axis represents the corresponding number of significant genes identified at the end of label propagation workflow.

**Gene**	**Functional Role**	**Pathways in PID**
GJA1	Cell growth, apoptosis, adhesion	AP-1 TF network; N-cadherin signaling events
RRM2	DNA repair	E2F TF network
TK1	DNA replication	E2F TF network; Validated targets of C-MYC transcriptional activation
MMP1	Invasion	AP-1 TF network; Syndecan-1-mediated signaling events
AURKB	Mitosis, cell cycle	Aurora A/B/C signaling; FOXM1 TF network
CCNB2	Mitosis, cell cycle	FOXM1 TF network;<br>Validated transcriptional targets of deltaNp63 isoforms
CDK1	Mitosis, cell cycle	AP-1 TF network; E2F TF network; FOXM1 TF network;<br>PLK1 signaling events; p73 TF network
PRC1	Mitosis, cell cycle	PLK1 signaling events
TPX2	Mitosis, cell cycle	Aurora A signaling; PLK1 signaling events
RRAGA	Signaling	mTOR signaling pathway

Supplementary Table S1: Tamoxifen signature and associated signaling events. The first column lists genes from this signature that formed the labeled node set. The second column lists annotations of their functional roles, determined by the original study. The third column lists pathways to which each gene belongs in the network obtained with label propagation (Figure 2b).

**Starting Set 1**	**Starting Set 2**	**MCS Distance**
Noncanonical Wnt signaling pathway & E2F transcription factor network & Regulation of nuclear SMAD2/3 signaling	**Noncanonical Wnt signaling pathway**& **E2F transcription factor network**&**Regulation of nuclear SMAD2/3 signaling** (2)	0.34
	**Noncanonical Wnt signaling pathway** & **E2F transcription factor network** & AP-1 transcription factor network	0.59
	**Noncanonical Wnt signaling pathway** & **Regulation of nuclear SMAD2/3 signaling** & p53 pathway	0.63
	**E2F transcription factor network** & **Regulation of nuclear SMAD2/3 signaling** & IL6-mediated signaling events	0.69
	**Noncanonical Wnt signaling pathway** & AP-1 transcription factor network & ATR signaling pathway	0.77
	**Regulation of nuclear SMAD2/3 signaling** & p73 transcription factor network & CXCR3-mediated signaling events	0.79
	**E2F transcription factor network** & PLK1 signaling events & p53 pathway	0.89
	CXCR3-mediated signaling events & ATR signaling pathway & p73 transcription factor network	0.92
	AP-1 transcription factor network & PLK1 signaling events & ATM pathway	0.96

Supplementary Table S2: MCS-based distance values obtained across a variety of network comparisons. First two columns list the names of the pathways that contribute to the labeled node sets used to generate the networks used in these tests. Bold pathway names in the second column represent the pathways that are also part of the "starting set 1" shown in the first column. The third column represents the value of distance metric when a given network listed in the second column is compared to the network obtained with starting set 1 seed gene set.
